# Supplementary material for: Mechanical architecture and folding of E. coli type 1 pilus domains
Source: Nat Commun. 2018 Jul 16;9:2758. doi: 10.1038/s41467-018-05107-6 (PMC6048123; doi:10.1038/s41467-018-05107-6)
Supplement: Supplementary file 1 — Supplementary Information [file 41467_2018_5107_MOESM1_ESM.pdf]

SUPPLEMENTARY INFORMATION

Mechanical architecture and folding of *E coli* type 1 pilus domains

Supplementary Note

Protein sequences

I91 protein sequence

LIEVEKPLYGVEVFVGETAHFEIELSEPDVHGQWKLKGQPLTASPDCEIIEDGKK
HILILHNCQLGMTGEVSFQAANAKSAANLKVKE

Self-complemented Fim proteins sequences

FimA-DS$_{FimA}$

ACAVDAGSVDQTVQLGQVRTASLAQEGATSSAVGFNIQLNDCDTNVASKAAV
AFLGTAIDAGHTNVLALQSSAAGSATNVGVQILDRTGAALTLDGATFSSETTLN
NGTNTIPFQARYFATGAATPGAANADATFKVQYQDNKQAATTVNGGTVHFKG
EVVNA

FimF-DS$_{FimA}$

DGNGCSVAAESTNFTVDLMENAAKQFNNIGATTPVVPFRILLSPCGNAVSAVK
VGFTGVADSHNANLLALENTVSAASGLGIQLLNEQQNQIPLNAPSSALSWTTLT
PGKPNTLNFYARLMATQVPVTAGHINATATFTLEYQDNKQAATTVNGGTVHF
KGEVVNA

FimG-DS$_{FimF}$

VVAKPCTVSTTNATVDLGDLYSFSLMSAGAASAWHDVALELTNCPVGTSRVT
ASFSGAADSTGYYKNQGTAQNIQLELQDDSGNTLNTGATKTVQVDDSSQSAHF
PLQVRALTVNGGATQGTIQAVISITYTYSDNKQADSTITIRGYVR

FimH$_L$-FimH$_P$-DS$_{FimG}$

FACKTANGTAIPIGGGSANVYVNLAPVVNVGQNLVVDLSTQIFCHNDYPETITD
YVTLQRGSAYGGVLSNFSGTVKYSGSSYPFPTTSETPRVVYNSRTDKPWPVAL
YLTPVSSAGGVAIKAGSLIAVLILRQTNNYNSDDFQFVWNIYANNDVVVPTGG
CDVSARDVTVTLPDYPGSVPIPLTVYCAKSQNLGYYLSGTTADAGNSIFTNTAS
FSPAQGVGVQLTRNGTIIPANNTVSLGAVGTSAVSLGLTANYARTGGQVTAGN
VQSIIGVTFVYQDNKQADVTITVNGKV

FimC

MSNKNVNVRKSQEITFCLLAGILMFMAMMVAGRAEAGVALGATRVIYPAGQK
QVQLAVTNNDENSTYLIQSWVENADGVKDGRFIVTPPLFAMKGKKENTLRILD
ATNNQLPQDRESLFWMNVKAIPSMDKSKLTENTLQLAIISRIKLYYRPAKLALP
PDQAAEKLRFRRSANSLTLINPTPYYLTVTELNAGTRVLENALVPPMGESTVKL
PSDAGSNITYRTINDYGALTPKMTGVME

DsbA

AQYEDGKQYTTLEKPVAGAPQVLEFFSFFCPHCYQFEEVLHISDNVKKKLPEGV
KMTKYHVNFMGGDLGKDLTQAWAVAMALGVEDKVTVPLFEGVQKTQTIRSA
SDIRDVFINAGIKGEEYDAAWNSFVVKSLVAQQEKAADVQLRGVPAMFVNG
KYQLNPQGMDTSNMDVFVQQYADTVKYLSEKK

Domain	Force (pN) ± SEM	Measured ΔL$_c$ (nm) ± SD	Theoretical ΔL$_c$ (nm)
FimA oxidized	527±6	42±2	44
FimA reduced	308±3	57±1	60
FimF oxidized	418±4	43±2	45
FimF reduced	272±4	55±1	61
FimG oxidized	431±4	40±2	39
FimG reduced	339±3	52±1	55
FimH$_P$ subdomain oxidized	362±6	38±1	39
FimH$_P$ subdomain reduced	235±11	47±2	50
FimH$_L$ subdomain<br>FimH$_L$ with intermediate	130±5<br>98±8<br>107±5	40±2<br>6±1<br>36±1	41

Supplementary Table 1. Summary of the unfolding forces and the increments of contour length (*ΔL$_c$*) of the Fim proteins in their oxidized and reduced state determined by smFS (FimA$_{S\text{-}S}$ n = 43, FimA$_{SH\text{-}SH}$ n = 68, FimF$_{S\text{-}S}$ n = 79, FimF$_{SH\text{-}SH}$ n = 40, FimG$_{S\text{-}S}$ n = 110, FimG$_{SH\text{-}SH}$ n = 65, FimH$_{PS\text{-}S}$ n = 51, FimH$_{PSH\text{-}SH}$ n = 12, FimH$_L$ n = 26, FimH$_L$ with intermediate n=16). Data shows the mean ± SEM for the unfolding forces (pN) and the mean ± SD for the increments of contour length (nm). Theoretical values for contour length are determined by multiplying the number of residues by 0.4 and subtracting the length of the folded domain[1]. The discrepancy between measured and theoretical ΔL_c comes from the fact that for theoretical calculations all residues are accounted. However, the actual mechanical clamp generally does not include all residues, as some of them in the termini may not have a real contribution to the mechanical stability.

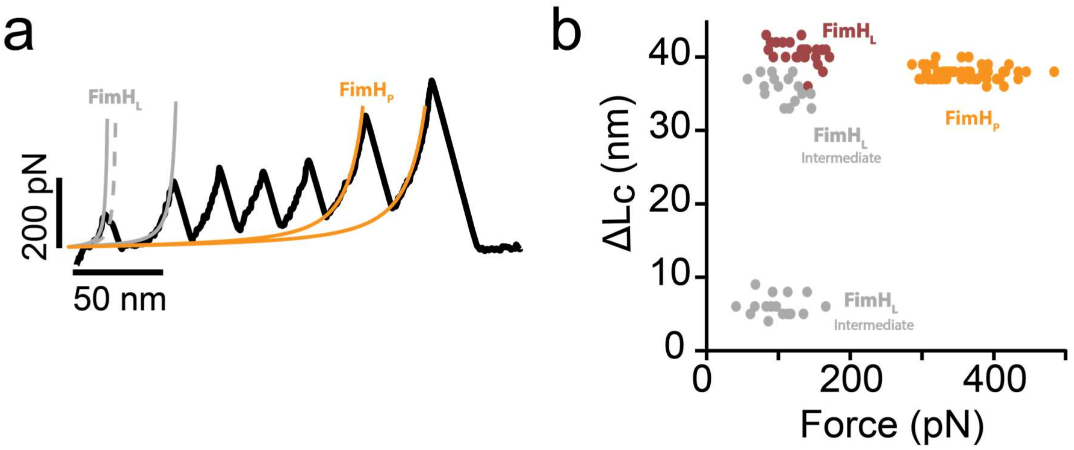

Supplementary Figure 1. FimH lectin subdomain intermediate. (**a**) Force-extension trace of FimH showing the unfolding of the FimH$_L$ through one intermediate. The grey lines represent the fitting of the unfolding of the FimH$_L$ subdomain with the worm-like chain model. The dotted line corresponds to the intermediate found in about one third of the total data. (**b**) Contour length increment (ΔL_c) *vs* force scatter plot of FimH showing the data of oxidized pilin subdomain FimH$_P$ in orange, 362±6 pN and 38±1 nm (n=51, total number of traces). The lectin subdomain in one step is represented by the brown dots at 40±2 nm and 130±5 pN (n = 26). This domains can unfold through an intermediate and two populations are monitored, large extension at 36±1 nm and 107±5 pN and short peak at 6±1 nm and 98±8 pN. This two populations are represented by the grey dots in the histogram (n=16). We acknowledge that in some traces only FimH$_P$ is detected.

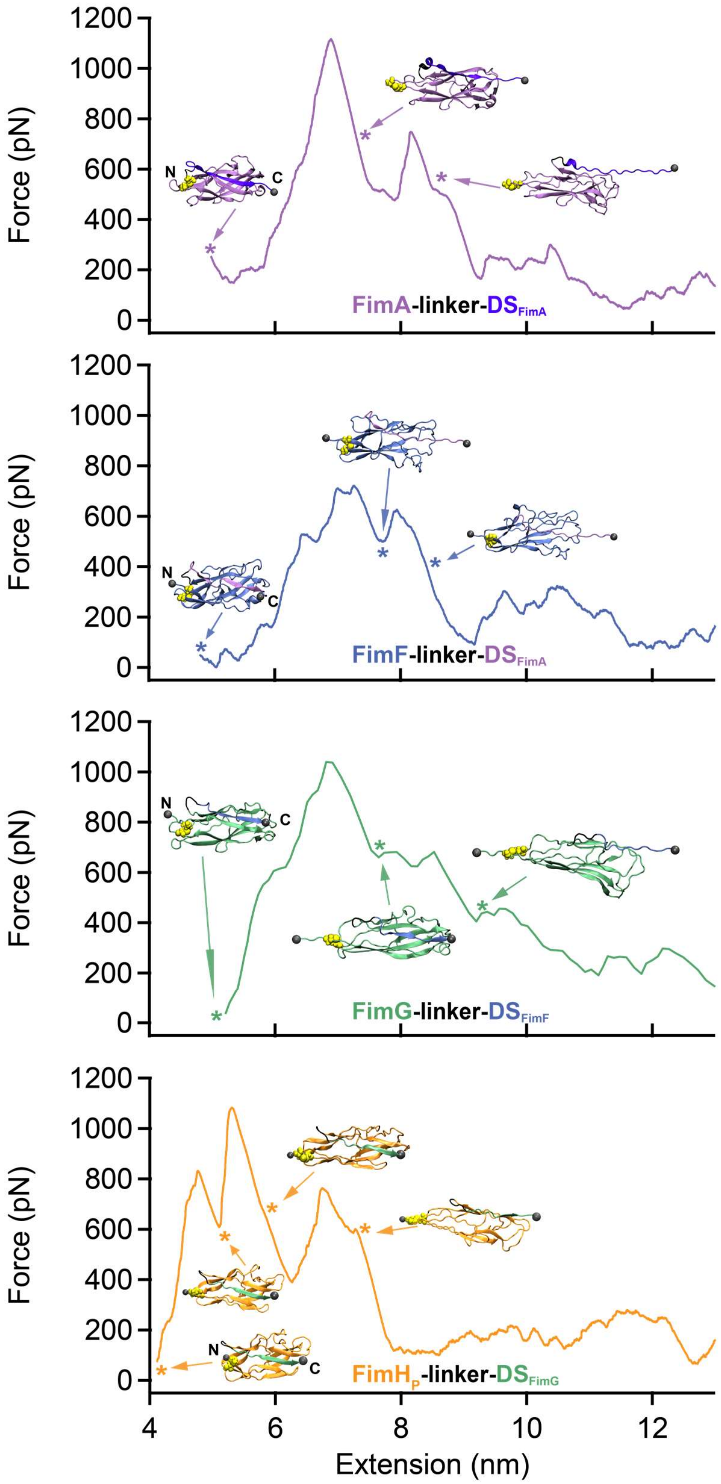

Supplementary Figure 2. Force *versus* Extension plots of the Steered Molecular Dynamics simulations for the self-complemented Fim proteins. From top to bottom we show FimA, FimF, FimG and FimH. The cartoon insets show the proteins at different extensions during the pulling. N and C terminal aminoacids are highlighted as grey beads, except for FimA whose N-terminal aminoacid is a cysteine, shown with a van der Waals representation. The four aminoacid linkers (DNKQ) are represented in black.

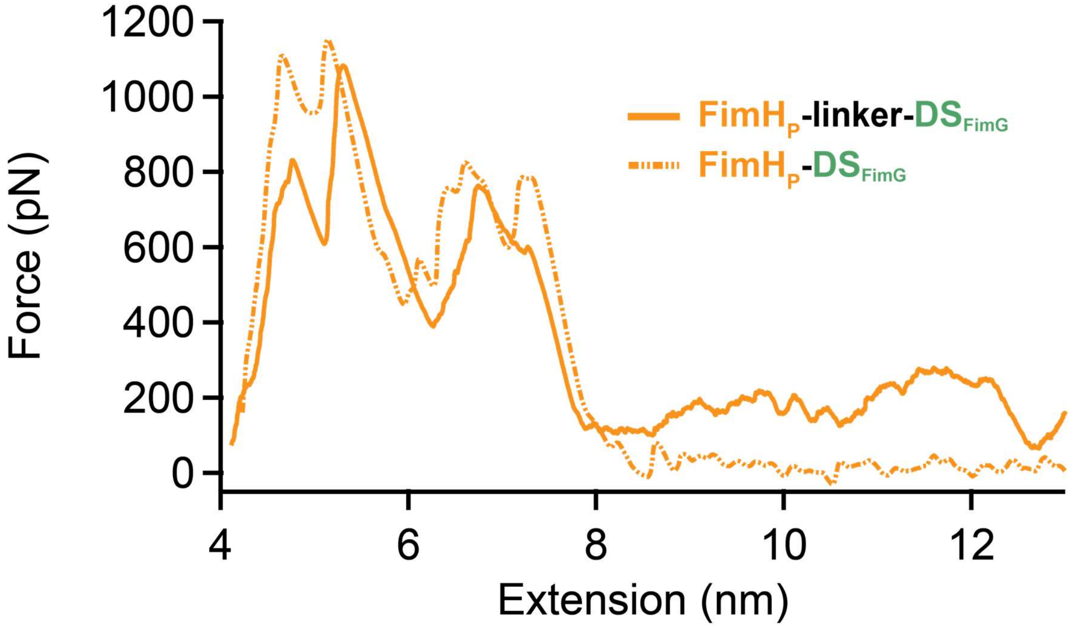

Supplementary Figure 3. Force *versus* extension of the Steered Molecular Dynamics simulations of the self-complemented FimH pilin domain and the FimH pilin domain with the donor β-strand of FimG. The self-complemented FimH shows the same unfolding pattern and the same mechanical stability as the FimH domain complemented with the donor β-strand of FimG.

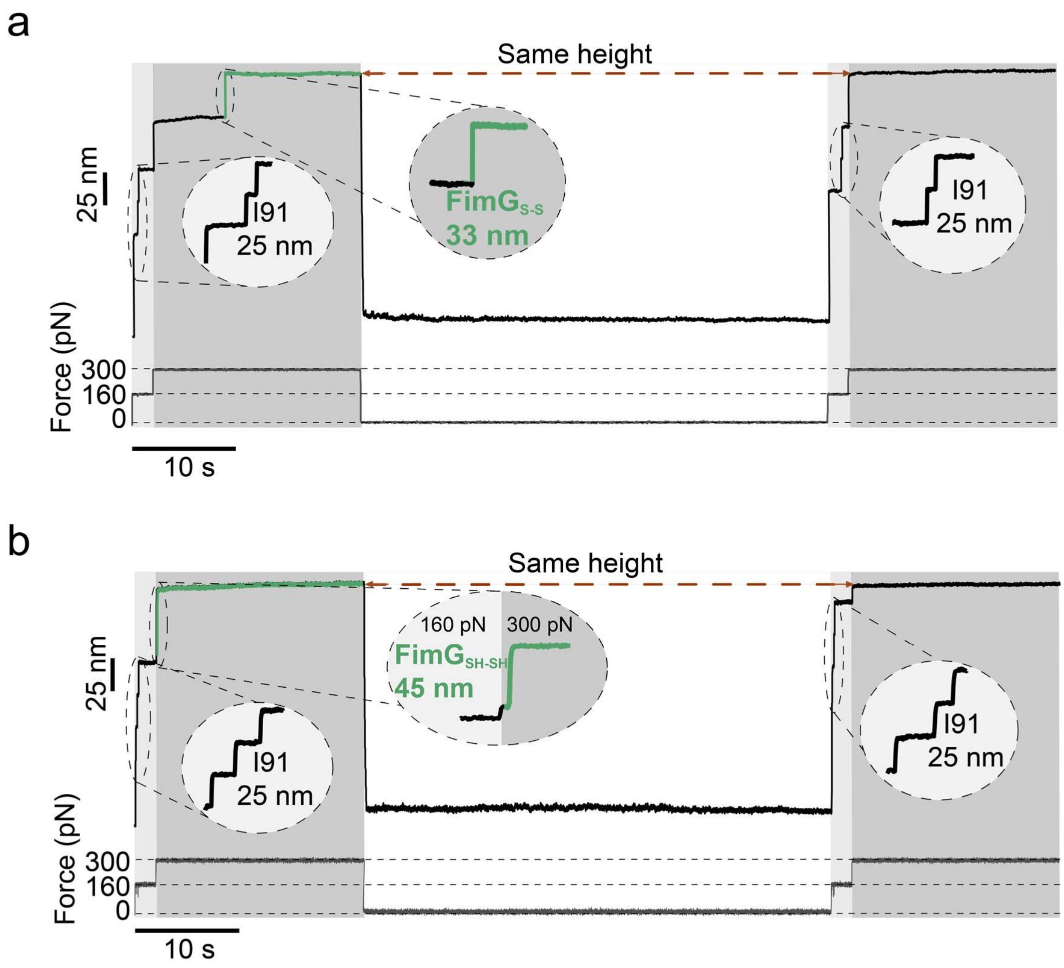

Supplementary Figure 4. Force-clamp refolding experiments of FimG. The figure shows two example traces from unfolding and refolding experiments of FimG at 45 s of quenching time. In each trace I91 unfoldings are detected as 25 nm increase in length, meanwhile FimG is detected as an increase of 33 nm (when disulfide-bonded, (**a**)) or 45 nm (reduced, (**b**)). Dotted brown line indicates the stretched molecule has the same height on both sides. The different gray scale of the shaded areas indicates the constant force applied along the experiment (grey, 160 pN for 2 s; dark grey, 300 pN for 20 s; and light grey, 0 pN for different times).

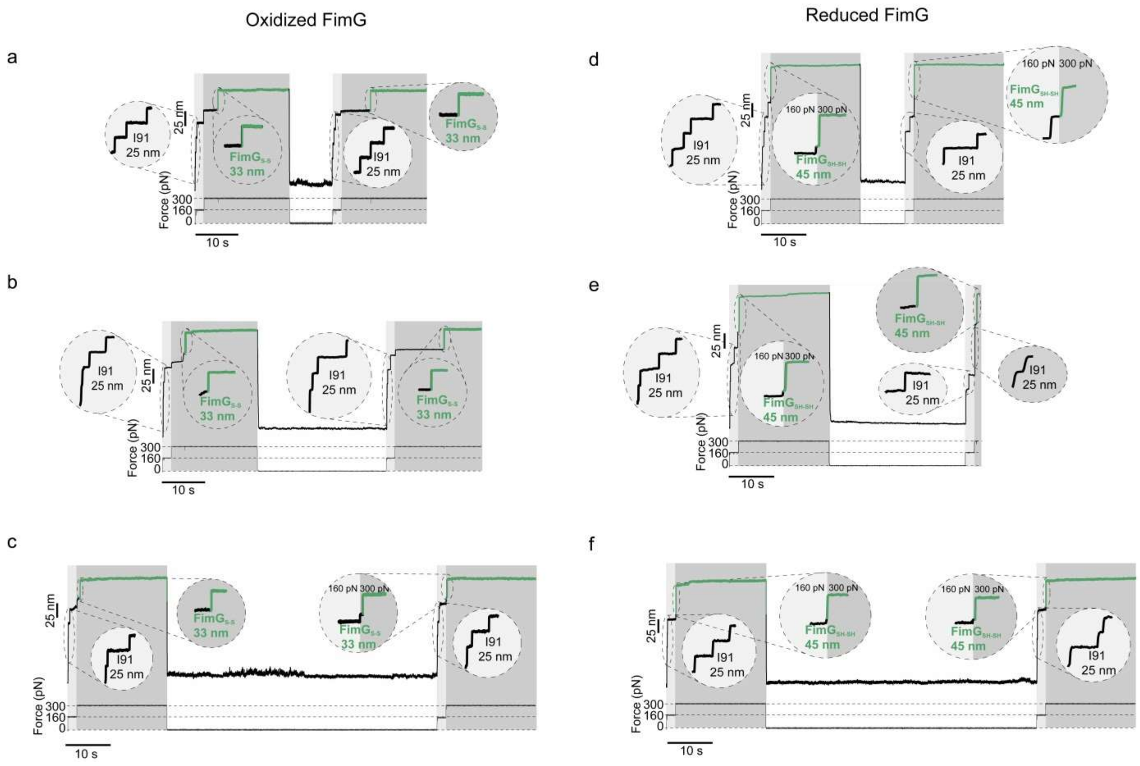

Supplementary Figure 5. Force-clamp refolding experiments of FimG in the presence of FimC. The figure shows six example traces from unfolding and refolding experiments of FimG in the presence of FimC at different quenching times. In each trace I91 unfoldings are detected as 25 nm increase in length, meanwhile FimG is detected as an increase of 33 nm (when disulfide-bonded) or 45 nm (reduced). Oxidized FimG (FimG$_{S\text{-}S}$) at 10 s (**a**), 30 s (**b**) and 60 s (**c**) of quenching time. Reduced FimG (FimG$_{SH\text{-}SH}$) at 10 s (**d**), 30 s (**e**) and 60 s (**f**) of quenching time. The different gray scale of the shaded areas indicates the constant force applied along the experiment (grey, 160 pN for 2 s; dark grey, 300 pN for 20 s; and light grey, 0 pN for different times).

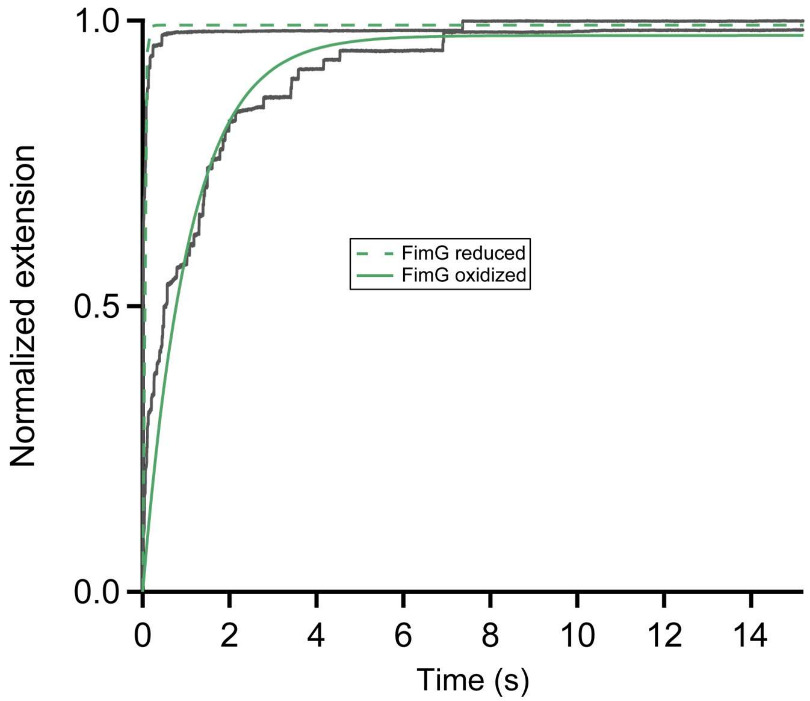

Supplementary Figure 6. Unfolding rate at 300 pN. Exponential fitting of the summed and averaged traces of the unfolding of oxidized $FimG_{S\text{-}S}$ (n=52) and reduced $FimG_{SH\text{-}SH}$ (n=58) at 300 pN. The measured rates are, FimG oxidized: $1/\tau_1$=0.94 s^{-1}; FimG reduced: $1/\tau_1$=29.9 s^{-1}).

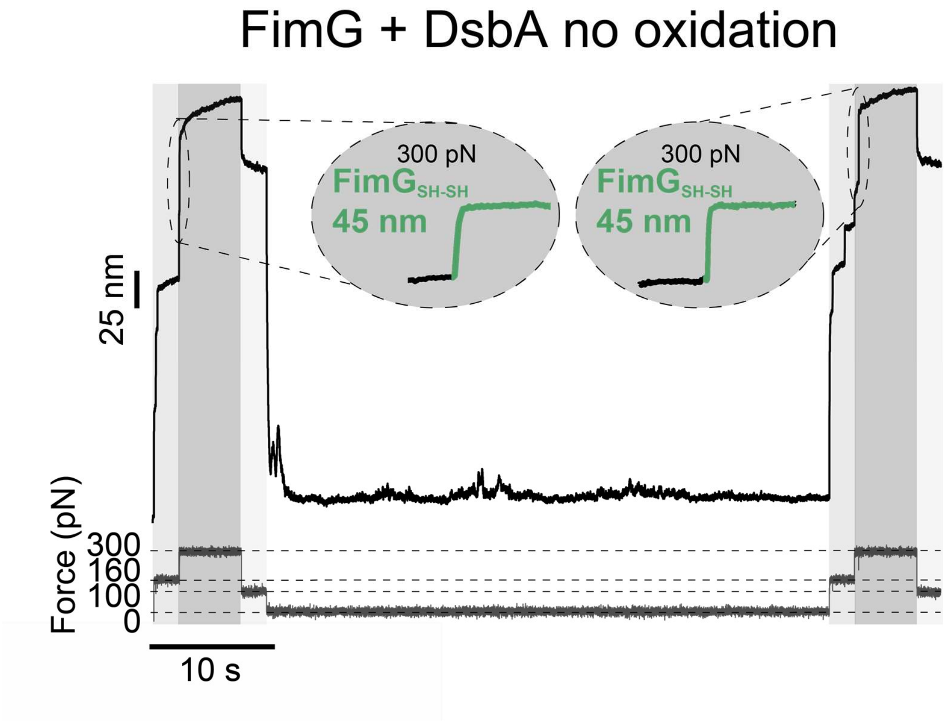

Supplementary Figure 7. Folding of reduced FimG in the presence of DsbA with no oxidase activity detected. Experimental force-clamp trace with no change in the oxidation state of FimG. The trace is a four-force protocol (160 pN, 300 pN, 100 pN, 0 pN) in which FimG is unfolded at 300 pN giving a step size corresponding to $FimG_{S-S}$. After the quench for 45 s, the same protocol is repeated rendering the exact same situation, which means that DsbA did not oxidize the disulfide bond in the substrate but still folds. We believe that there is a population of reduced DsbA able to reduce FimG. However, even in that situation we did not observe oxidase activity given that the disulfide bond of FimG remained reduced. Again, we still oberved folding of FimG.

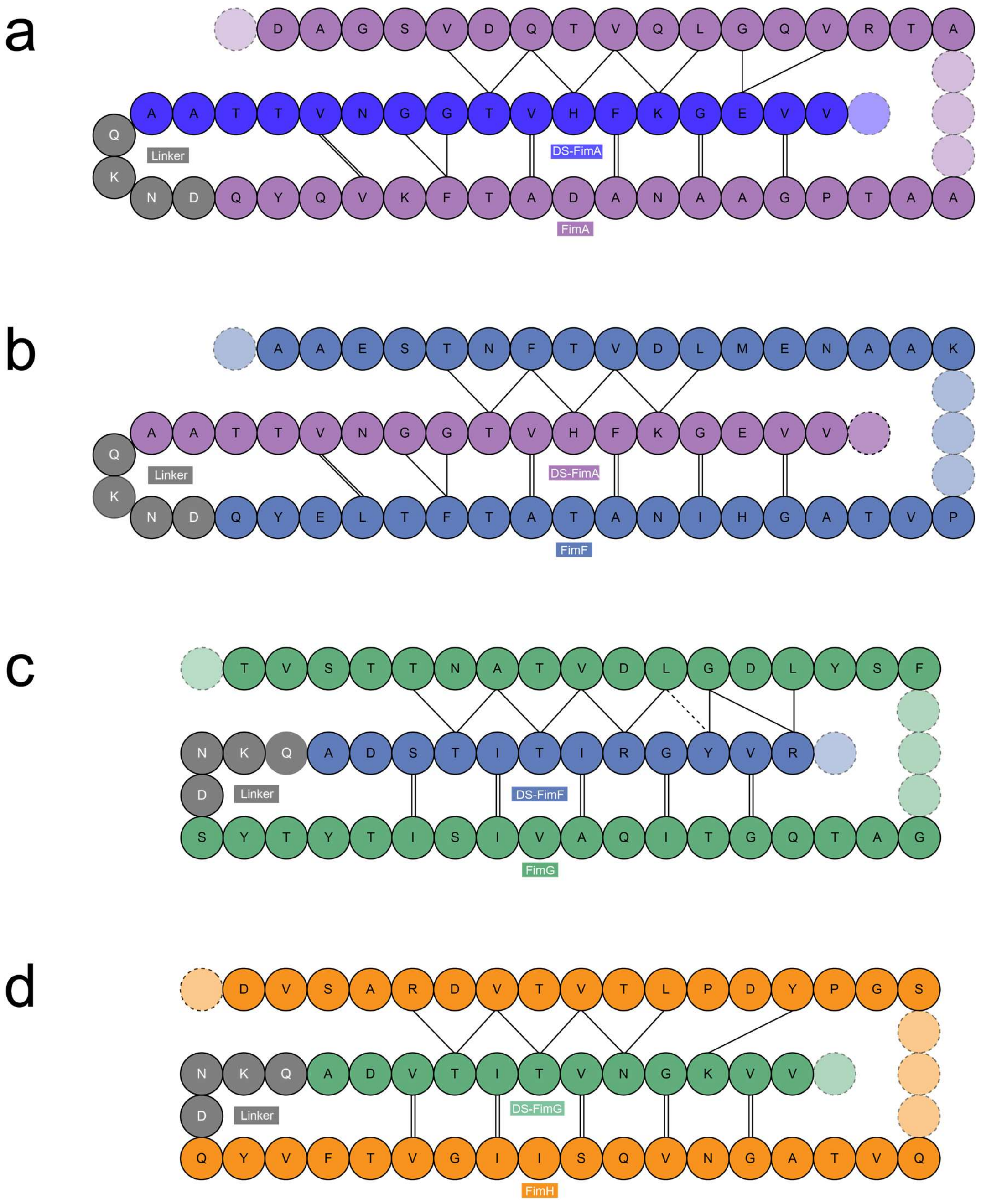

Supplementary Figure 8. Hydrogen bond pattern of the self-complemented Fim proteins. The bonding pattern is recovered by examining the fluctuation of O-H bond lengths in the equilibrated structure before the start of the pulling. Each circle represents a residue, and a single line connecting two circles shows a hydrogen bond between them. Double lines indicate two hydrogen bonds within the same pair of residues. The dotted line indicates a weak bond. Within each protein, the top strand and donor strand exhibit parallel β-sheet bonding (given by the zig-zag bonding pattern), while the bottom strand and donor strand exhibit antiparallel β-sheet bonding (given by the row of double bonds).

References

1 Ainavarapu, S. R. *et al.* Contour length and refolding rate of a small protein controlled by engineered disulfide bonds. *Biophys J* **92**, 225-233, (2007).
